# N-Acetylcysteine Attenuates Cisplatin Toxicity in the Cerebrum and Lung of Young Rats with Artificially Induced Protein Deficiency

**DOI:** 10.3390/ijms25116239

**Published:** 2024-06-05

**Authors:** David Calderón Guzmán, Norma Osnaya Brizuela, Maribel Ortíz Herrera, Armando Valenzuela Peraza, Norma Labra Ruíz, Hugo Juárez Olguín, Daniel Santamaria del Angel, Gerardo Barragán Mejía

**Affiliations:** 1 Laboratory of Neurosciences, Instituto Nacional de Pediatria (INP), Mexico City 04530, Mexico; davidcaguama91@gmail.com (D.C.G.); osnayanorma@hotmail.com (N.O.B.); valenzuela.peraza2013@hotmail.com (A.V.P.); norma_labra@hotmail.com (N.L.R.); santamaria.daniel@gmail.com (D.S.d.A.); 2 Laboratory of Experimental Bacteriology, Instituto Nacional de Pediatria INP, Mexico City 04530, Mexico; mortizherrera@hotmail.com (M.O.H.); mg_barragan@hotmail.com (G.B.M.); 3 Laboratory of Pharmacology, Instituto Nacional de Pediatría, Avenida Imán N° 1, 3rd piso Colonia Cuicuilco, Mexico City 04530, Mexico

**Keywords:** antioxidant biomarkers, cisplatin, N-acetylcysteine, glutathione, malnutrition

## Abstract

Neurotoxicity is a major obstacle in the effectiveness of Cisplatin in cancer chemotherapy. In this process, oxidative stress and inflammation are considered to be the main mechanisms involved in brain and lung toxicity. The aim of the present work was to study the influence of the amount of protein on some oxidative parameters in the brain and lungs of rats treated with Cisplatin (CP) and N-Acetylcysteine (NAC) as neuroprotectors. Four groups of Wistar rats, each containing six animals, were fed with a protein diet at 7% for 15 days. Thereafter, the groups were given either a unique dose of CP^®^ 5 mg/kg or NAC^®^ 5 mg/kg as follows: group 1 (control), NaCl 0.9% vehicle; group 2, CP; group 3, NAC; and group 4, NAC + CP. The animals were sacrificed immediately after the treatments. Blood samples were collected upon sacrifice and used to measure blood triglycerides and glucose. The brain and lungs of each animal were obtained and used to assay lipid peroxidation (TBARS), glutathione (GSH), serotonin metabolite (5-HIAA), catalase, and the activity of Ca^+2^, and Mg^+2^ ATPase using validated methods. TBARS, H_2_O_2_, and GSH were found to be significantly decreased in the cortex and cerebellum/medulla oblongata of the groups treated with CP and NAC. The total ATPase showed a significant increase in the lung and cerebellum/medulla oblongata, while 5-HIAA showed the same tendency in the cortex of the same group of animals. The increase in 5-HIAA and ATPase during NAC and CP administration resulted in brain protection. This effect could be even more powerful when membrane fluidity is increased, thus proving the efficacy of combined NAC and CP drug therapy, which appears to be a promising strategy for future chemotherapy in malnourished patients.

## 1. Introduction

The cancer death toll around the world has toppled all other causes leading to the loss of life. The annual new cases, due to this ailment, have surpassed ten million people, while the death registry is estimated to be over six million. Yildiz et al. [1] found that more than 80% of cancers from which the people suffer are preventable, and this can be achieved by good nutrition. Falls among older people are common and associated with substantial morbidity, mortality, and healthcare costs. In the United States, malignant tumors are currently the main cause of death in children under 15 years of age [2]. Cancer and its treatments potentiate important risk factor for falls, including muscle weakness, poor balance, proprioception, cognitive impairment, and functional disability.

In the management of cancer, chemotherapy has been a useful tool. However, it produces an increase in the accumulation of monoamine in the brain [3], and neurotransmitters can influence immune cells and endothelial cells in the tumor microenvironment to promote tumor progression [4]. However, the crucial role of monoamine oxidases (MAOs) is to maintain functional levels of neurotransmitters, and implications of distortions in its activity have been associated with tumor progression [5].

Cisplatin (CP) is a chemotherapeutic agent that is frequently employed in cancer treatment [6]. In fact, it still remains the only therapeutic option for several tumors; however, its demerits lie in the fact that it is associated with the induction of side effects [7]. In this regard, the drug has been shown to have a significant association with increased triglyceride levels as well as higher fluctuations in the level of lipids in the blood serum [8]. Indeed, to achieve the desired pharmacological effect, only a very small amount of such a catalytic metallodrug is required [9].

In cancer treatment, CP eliminates cancer cells at some points of development and growth [10]. This action has been linked to its ability to crosslink with the purine bases in the DNA; thus, it interferes with the DNA repair mechanisms, and causes the damage and apoptosis of cells [11]. The role of mitochondrial DNA alterations in the onset of resistance phenomena is related both to redox balance alterations [12] and the dysfunction of the mitochondrial respiratory chain induced by CP, which results in the overproduction of reactive oxygen species (ROS) [13]. Therefore, when CP enters the cells, they become vulnerable to cytoplasmic inactivation by GSH (an important endogenous nucleophile) [14]. Hence, the challenge of new drugs in cancer treatment is to reduce their neurotoxic and other side effects.

Chemotherapy in young children, apart from inducing negative effects, produces malnutrition complications [15]. In this respect, protein malnutrition (PM), together with its concomitant compromise of the antioxidant defense system of the body, can be highlighted. Glucose deficiency involves an insufficient amino acid supply [16]. However, malnourished patients who received antioxidant supplementation as NAC have been found to have considerable clinical benefits [17]. Currently, this supplement is widely included in chemotherapy protocols, even when some studies have suggested the effective function of externally derived polyunsaturated fatty acids (PUFAs) in the modulation of the cytotoxicity of anti-cancer drugs [18]. CP, a chemotherapeutic drug, executes action mechanisms on cell apoptosis and ferroptosis by disrupting the glutathione (GSH) metabolism. In addition, it executes mitochondria-mediated apoptosis and lipid oxidation-related ferroptosis through activating IL6/JAK/STAT3 signaling [19]. Free radicals (FR) produce detrimental effects on cell organelles [20], especially the lipid constituent of the plasma membranes [21]. Fortunately, the central nervous system (CNS) mediates not only the consumption of food but also the production of FR and plays an active role in food metabolism [22]. The cell membrane is composed of different types of lipids, and modifications in this cell structure can affect many biological processes [23]. In the brain, the phospholipids that compose the plasma membrane are contiguous with the protein architecture inside the double-strand lipid layers of the membrane [24]. The interchange of ions taking place in these double-strand lipid layers is facilitated by Na^+^, the K^+^ ATPase enzyme which stimulates the entrance and exit of Na^+^ and K^+^ in the cell [25]. In the CNS, when the action of ATPase is inhibited, there is an induction of the release of excitatory amino acids, such as serotonin [26]. Therefore, it is of paramount importance to find a treatment that can synergistically attenuate the toxic effects of most cancer drugs to ensure optimum benefit and tolerance of their use.

In view of the information cited in the paragraphs above, this work aimed to assess the level of protection that the NAC treatment offered to rats in which protein deficiency had been artificially produced with the CP treatment. This work performed a comparative analysis of the protection benefits of NAC in the maintenance of the lung and the levels of 5-HIAA monoamine, GSH, catalase, total ATPase, and TBARS in the cortex, hemispheres, and cerebellum of animals treated with CP.

## 2. Methods

### 2.1. Experimental Animals

The twenty-four young male Wistar rats used in this study were purchased from the certified laboratory animal breeding house of Instituto Politécnico Nacional, Mexico City.

The animals were placed in four meshed plastic cages, each containing six rats. They were exposed to both light and darkness, each with a duration of 12 h, in natural environmental conditions. An acclimatization period of 1–2 weeks to the animal house facility conditions with food and water was allowed for the rats. Following this period, the animals were fed with granulated laboratory rodent feed containing 23% of protein (Purine 5001^®^), with no restrictions. Water was freely allowed during the experiment.

### 2.2. Chemicals

Thiobarbituric acid (TBA), glutathione, catalase, ATP, and 5-HIAA were obtained from Sigma-Aldrich, St. Louis, MO, USA. Hydrochloric acid, sulfuric acid, nitric acid, bisulfite, trichloro acetic acid, sodium phosphate, magnesium chloride, and Tris-HCl were purchased from Merck, Darmstad, Germany.

### 2.3. Experimental Drugs

Twenty-four young male Wistar rats, each weighing 200 g, were employed in this study. The rats were indiscriminately assigned to a group. Each group consisted of six animals, making up a total of four groups. Every animal in each group was fed with a protein diet at 7% for 15 days [27]. Thereafter, and based on the group, the animals were treated either with CP^®^ at 5 mg per kg weight (unique dose) or NAC^®^ at 5 mg per kg weight (unique dose). The administration of the treatments was intraperitoneal and performed in the following way: A vehicle of NaCl at 0.9% was administered to the control animals corresponding to group 1. CP was given to the animals assigned to group 2. The rats that formed group 3 received only NAC, while those which formed group 4 were administered NAC + CP, with a 60 min interval between each treatment. Hellberg et al. [28] found that the CP serum concentration after 30 min of administration is the same as that after 60 min.

The sacrifice of the rats was performed sixty minutes after drug administration by decapitation and without anesthesia. During the sacrifice, animal blood was obtained and used to determine lipid peroxidation (TBARS), serotonin metabolite (5-HIAA), glutathione (GSH), catalase, and Ca^+2^ and Mg^+2^ ATPase activities. Also, a portion of the animal brain and lung was extracted immediately after the sacrifice and employed to assay Ca^+2^, Mg^+2^ ATPase, TBARS, 5-HIAA, GSH, and catalase. For the assays, the extracted brain tissues were dissected and separated into cortex, hemispheres, and cerebellum [29]. These brain structures were stored in a sodium chloride (NaCl) solution with a concentration of 0.9%. Later, the structures and the lungs were homogenized in 3 mL of tris-HCl 0.05 M, pH 7.2. Subsequently, the homogenates were preserved at –80 °C until analyzed. The procedures to perform the experiments were in accordance with the national and international standard procedures for the care and use of laboratory animals. The Ethics Committee of Instituto Nacional de Pediatria gave its approval to this research protocol with reference number 026/2022. Moreover, all the experiments were performed in accordance with the relevant guidelines and regulations.

### 2.4. Brain Structure Dissection

The rhombencephalon was separated from the rest of the brain by a transverse section. This was dissected into the cerebellum and the medulla oblongata. A transverse section was made at the level of the optic chiasma, which delimits the anterior part of the hypothalamus and passes through the anterior commissure. This section separates the brain into two parts—cortex and hemispheres. This procedure was made with stereotaxic devices.

### 2.5. Glucose and Triglyceride Assessment

The levels of triglycerides and glucose were measured with 20 µL of fresh blood using a previously calibrated test strip with Roche devices.

### 2.6. 5-HIAA Assay and Assessment

The assessment of 5-hydroxyindol acetic acid was performed using the supernatant obtained from the brain and lung tissue sections that were homogenized in perchloric acid (HCLO_4_) and centrifuged at 9000 rpm for 10 min in a microcentrifuge (Hettich Zentrifugen, model Mikro 12-42, Tuttlingen, Germany), with a modified version of the technique reported by Beck et al. [30] and modified by Peraza et al. [31]. A portion of the supernatant was taken and put in a test tube containing 1.9 milliliters of a 0.1 M acetate buffer at pH 5.5. A five-minute incubation of this mixture was performed at room temperature in total darkness. Thereafter, each sample was spectrofluorometrically read (Perkin Elmer LS 55, Beaconsfield, UK) at emission and excitation lengths of 333 nm and 296 nm, respectively. The software employed in this assessment was 4.00.02 version of FL Win Lab. Using a curve which had already been standardized, the inference of 5-HIAA values was made and recorded in nM/g of wet tissue.

### 2.7. GSH Assessment

Glutathione measurement was performed with the supernatant of the brain and lung tissue sections as described for 5-HIAA and in accordance with the technique of Hissin and Hilf [32] and modified by Peraza et al. [31]. In a test tube, a mixture containing the supernatant (20 μL), ortho-phthaldehyde in methanol (100 mL at 1 mg/mL), and phosphate buffer (1.8 mL) with pH of 8.0 and EDTA 0.2% was prepared. A fifteen-minute incubation of this mixture was carried out at room temperature in absolute darkness. The PERKIN ELMER LS 55 spectrophotometer, with excitation 350 and emission 420 wavelengths, was used to assess the level of GSH. The software used in this assessment was 4.00.02 version of FL Win Lab. In an already standardized curve, inference of the GSH values was carried out and recorded as nM/g tissue.

### 2.8. Measurement of Catalase (CAT)

Catalase (CAT) activity in hemispheres, cortex, and medulla/oblongata was assessed with the modified version of the technique of Hadwan [33] and reported in µmoles of H_2_O_2_ degraded/g tissue.

### 2.9. Total ATPase Assessment

The assay of ATPase was performed using Guzman et al.’s technique [34]. From the tris-HCl 0.05 M pH 7.4 homogenized tissues of hemispheres, cortex, and medulla/oblongata, 1 mg (10%) *w*/*v* was taken and added to a solution containing sodium chloride (100 mM), potassium chloride (7 mM), and magnesium chloride (13 mM). Tris-ATP (4 mM) was added to the solution. In a shaking water bath (Dubnoff Labconco, IL, USA), the resultant solution was subjected to 30 min incubation at a temperature of 37 °C. The reaction was stopped using 100 µL of trichloroacetic acid *w*/*v* at a concentration of 10%. Thereafter, 5 min centrifugation of the solution was carried out at 100× *g* under a temperature of 4 °C. With an aliquot of the supernatant and in accordance with Fiske and Subarrow’s method [35], the measurement of inorganic phosphate (Pi) was made in duplicates. The supernatant’s absorbance was spectrophotometrically read at 660 nm using Helios-α, UNICAM. The activity of the total ATPase was expressed as mM Pi/g wet tissue/per minute.

### 2.10. Assessment of Lipid Peroxidation

TBARS was assessed with Gutteridge and Halliwell [21]’s technique after modification and according to the works of Guzman et al. [34]. For this assessment, 1 mL of the tissues of hemispheres, cortex, and medulla/oblongata that had been subjected to homogenization in tris-HCl 0.05 M pH 7.4 was mixed in a 2 mL TBA solution. This solution contained TBA (1.25 g), a mixture of concentrated HCL (6.25 mL) and deionized water (250 mL), and trichloroacetic acid (TCA) (40 g). The solution was subjected to 30 min of heating until reaching a boiling point (Thermomix 1420, Braun, Melsungen, Germany). Thereafter, the solution was cooled for 5 min in an ice bath. It underwent 15 min of centrifugation at 700× *g* (Sorvall RC-5B Dupont, CA, USA). To determine the TBARS concentration, the absorbances of the brain tissue supernatants were spectrophotometrically interpreted in triplicate at 532 nm using Heλios-α of UNICAM. The concentration of substances reactive to Thiobarbaturic acid (TBARS) was expressed as µM of Malondialdehyde/g of wet tissue.

### 2.11. Statistical Analysis

The strategy for the inference analysis consisted in the comparison of the biochemical indicators between the control group and the different experimental groups, using some tests to contrast hypotheses—analysis of variance (Anova) or Kruskal–Wallis—prior to the verification of variance homogeneity. Post hoc contrasts were performed with Tukey–Kramer or Steel-Dwass tests. Any associated probability value α < 0.05 was considered statistically significant. The analysis was performed using the SAS Systems JMP v12 software for academic use.

## 3. Results

### 3.1. Glucose

The administration of CP increased the glucose levels in the animals of this group compared to the control group; however, this increase was not statistically significant. In the group treated with NAC + CP, a reduction in the concentration of this substance was observed in comparison with the rest of the experimental groups. This reduction was significant only when compared with the group treated with CP, ** *p* = 0.0009.

### 3.2. Triglycerides

There was an increase in the triglyceride levels in the groups treated with NAC alone or in combination with CP. However, the statistical analysis did not reveal significant differences (Table 1).

### 3.3. Lipid Peroxidation (TBARS)

In the groups of animals treated with CP and NAC + CP, a reduction in the concentration of Malondialdehyde was observed in the cortex (* *p* < 0.02) and hemispheres (* *p* < 0.01) when compared with both the control group and the NAC group. In the cerebellum, the reduction was significant only between the control group and the CP/NAC+ CP groups (* *p* = 0.04) (Table 2).

### 3.4. GSH

The GSH concentration showed a decrease in the cortex of the animals treated with NAC + CP with respect to the rest of the groups. This decrease was, however, statistically significant (* *p* = 0.01) when compared only with the group to which NAC had been administered. In the hemispheres, there was a GSH increase in the NAC + CP group, but the statistical analysis did not reveal significant differences. In the cerebellum, a decrease in GSH was observed in all the experimental groups; but, again, this decrease did not reach statistical significance (Table 3).

### 3.5. H_2_O_2_

In the cortex of the animals treated with NAC, CP, or NAC + CP, a decrease in H_2_O_2_ concentration was registered when the concentration of bioamine was compared with the animals in the control group. However, when the concentration was compared among the groups, there was a significant difference only when the treated groups were compared with the control group and NAC (* *p* = 0.02). On the contrary, an insignificant increase in H_2_O_2_ was registered in the hemispheres of the CP group when compared with the control group (* *p =* 0.02) and the group treated with NAC + CP (** *p* = 0.001). No significant difference was observed in the cerebellum (Table 4).

### 3.6. ATPase

The activity of the ATPase enzyme registered a slight increase in the cortex of the experimental animals with respect to the control group, but this did not reach statistical significance. In the hemispheres, the highest ATPase activity was observed with a decrease in activity in the NAC+ CP group when compared with the control group; nonetheless, the statistical difference was not significant. In the cerebellum, the administration of NAC, CP, and NAC+ CP produced a significant increase in ATPase activity (* *p* = 0.02) vs. NAC, (* *p* = 0.03) vs. CP, and (*p* = 0.004) vs. NAC + CP; (Table 5).

### 3.7. 5-HIAA

The levels of 5-HIAA in the cortex showed an increase in the animals that received NAC, CP, and NAC+ CP. The comparison between the control group and the group treated with NAC was statistically significant (** *p =* 0.007), while, between the CP/NAC+ CP groups and the control, no significant difference was observed.

In the hemispheres, the CP and NAC+ CP groups registered a decrease in 5HIAA levels, and only between the NAC and CP groups was this decrease statistically significant (* *p* = 0.02). In the cerebellum, no effect of the treatments on the 5HIAA levels was observed, (Table 6).

### 3.8. LUNG

The results of the biochemical indicators evaluated in the lung showed that the levels of lipid peroxidation (TBARS) had a slight decrease in the groups treated with NAC and CP, and a slight increase in the group treated with NAC + CP with respect to the control group, without these variations being statistically significant (*p* = 0.07). The administration of CP and NAC + CP produced an increase in the concentration of GSH when compared with the control and NAC groups; however, this increase did not reach statistical significance (*p* = 0.06). No effect of the treatments on the concentration of H_2_O_2_ in the lungs of the experimental animals was observed. Although there was an increase in the level of 5HIAA in the group treated with NAC + CP with respect to the level of this indolamine in the control group, the difference did not reach statistical significance (*p* = 0.08). The activity of the ATPase enzyme in the lung increased significantly in the group of animals that were administered NAC+ CP when compared with that of the control groups (** *p* = 0.005) and those treated with CP (* *p* = 0.01) (Table 7).

## 4. Discussion

One of the common adverse effects of oncologic drugs is neurological dysfunction.

Targeted therapies for cancer have been developed, and these are based on drugs that have significant metabolic consequences, which are associated with a significant increase in plasma triglycerides by reducing the activity of the lipoprotein lipase, in charge of the catabolism of triglyceride-rich lipoproteins and associated with a high incidence of hyperglycemia or hypoglycemia [36]. In this work, the use of CP in the treatment of the young animals, fed with a protein-deficient diet, decreased their lipid peroxidation levels, glutathione (GSH), and hydrogen peroxide (H_2_O_2_) in the cerebellum, cortex, and medulla oblongata.

The animal model employed were subjected to diet restriction, and, according to the literature, it is a suitable model to study how to maintain antioxidant status and tissue GSH re-establishment in severely compromised patients undergoing pharmacotherapy [37].

These experimental strategies suggest that the mechanism of resistance to CP from the perspective of the glucose metabolism suggests that SKOV3/DDP cells (cancer cell lines) exhibit decreased dependence on aerobic glycolysis and increased demand for glucose; thus, resetting the redox balance through the overexpression of the key glucose enzyme, glucose 6-phosphate dehydrogenase (G6PD), of the pentose phosphate pathway to eliminate the cytotoxicity of highly elevated ROS [38]. These metabolic disorders constitute a big metabolic brain and lung insult and induce oxidative damage. Sometimes, the cortex is involved. However, the lung and the cerebellum or brainstem are universally spared in this hyperglycemic and hypoglycemic damage. The reduction in Malondialdehyde in the tissues of animals treated with CP showed a significant association with the increase in triglyceride levels [8]. This result means that CP can alter the TBARs levels. In combination with NAC, the toxic effects of CP became mild [39]. While the increase in ATPase in the cerebellum and 5-HIAA in the cerebellum, cortex, and medulla oblongata indicates a reduction in oxidative stress due to the administration of NAC. This finding suggests that indolamines show protective effects on the toxic effects of CP in the same doses evaluated by Peng et al. [40]. In fact, the ATPase ion pump is responsible for the secretion of cerebrospinal fluid from the choroid plexus and is inhibited by serotonin via the stimulation of protein kinase C-catalyzed phosphorylation [41].

On the other hand, the increase in ATPase in the lungs of animals treated with NAC+ CP and in the cerebellum with the administration of NAC and NAC+ CP indicates that NAC can stop the adverse effects induced by CP. In fact, five cysteinyl residues (C452, C456, C457, C577, and C656) have been identified as the CP-binding sites on the cytoplasmic loop connecting transmembrane helices 4 and 5 (C45), inducing mutagenesis [42]. These residues are known to be susceptible to glutathionylation.

These findings are interesting, since CP has been shown to be cytotoxic to glioma cells. However, in animals, there are substantial limitations since it is very difficult for CP to penetrate across the blood–brain barrier (BBB). This problem can be resolved by the administration of a messenger drug with the ability to induce transient disruption of the BBB after 20 min of administration [43]. CP is a member of a widely utilized class of chemotherapeutic agents that induce DNA damage response, cell cycle arrest, and p53-dependent apoptotic cell death in concert with DNA–platinum adduct formation and normal programmed cell death (PCD) as oxidative damage [44].

The mitochondria are key regulators of cell survival, such as metabolism, Ca^2+^ signaling, and reactive oxygen species (ROS) production. However, ROS overproduction and mitochondrial Ca^2+^ overload are linked to the induction of apoptosis, while the impairment of mitochondrial dynamics and metabolism can have a double-faceted role in the decision between cell survival and death [45]. Indeed, chemoresistance, which may be due to the cooperation of several cellular protection mechanisms, is often mitochondria-related. Probably, the mitochondrial injury caused by CP can also be reversed, as evidenced in the improvement of mitochondrial morphology, the restoration of mitochondrial DNA content, and the reversal of mitochondrial gene expression [46].

On the other hand, Backway et al. suggest that chemotherapy agent-induced toxicity is accompanied by the loss of GSH, which leads to the induction of oxidative stress. According to the authors, the process seems to be a biphasic phenomenon where a state of highly reactive oxygen generation precedes permeability transition that occurs due to GSH depletion [47]. In the study performed by Tchantchou et al. [48], it was found that NAC is a cell-permeant antioxidant and glutathione precursor and that its administration as a supplement at 1 g/kg relieved oxidative damage and cognitive deterioration and re-established glutathione synthase and GSH levels [49], and lung GSH increased in the group that received NAC, while TBARS decreased in animals with a protein-deficient diet. These events coincide with the findings of Li and cols and suggest that cysteine prodrugs have the ability to restore GSH levels and that the redox status is tissue-specific [50]. Likewise, an insufficient amino acid supply promotes catabolic processes to aid cell survival [16]. Different cancer cell types may undergo different bioenergetic changes, some to a more glycolytic state and some to a more oxidative one. The energy powerhouse of the cell represents the key intracellular signaling hub that is emerging as an important determinant of several aspects of cancer development and progression, including metabolic reprogramming, the acquisition of metastatic capability, and response to chemotherapeutic drugs [51].

The mitochondria play an important role in effective cell energy production and cell survival under stress conditions, such as that elicited by treatment with chemotherapeutic drugs. Mitochondrial biogenesis, which is accompanied by the alteration in the mitochondrial energy metabolism, structure, and dynamics, indicates the important role of mitochondrial oxidative phosphorylation in CP resistance [52]. Indeed, mitochondrial stress-induced ROS production, as a feedback signal from the mitochondria to the cell nucleus, increases PGC1α expression in the cells, a process which is involved in mitochondrial oxidative phosphorylation regulation. Hence, adjunctive therapy based on dietary supplementation, which would facilitate the improvement of antioxidant status and the re-establishment of tissue GSH, may be developed. This would replace the expensive high-protein diet currently employed for this purpose in patients subjected to CP chemotherapy.

Oxygen is an important element in keeping the viability of neuronal cells. Nevertheless, neuronal cells have relatively fragile protective antioxidant mechanisms. Therefore, the regulation of the pro-oxidant–antioxidant balance could provide a therapeutic option, which can be used to improve neuroprotection in response to oxidative stress [53]. In fact, tumor cells often develop tolerance mechanisms by effectively repairing Cisplatin-induced DNA lesions or tolerating the damage by adopting translesion DNA synthesis. In addition, the treatment regimen can be improved by combining CP with certain molecular targeted therapies to achieve a balance between tumor toxicity and tolerance mechanisms [54]. Meanwhile, pyruvate kinase M2 (PKM2) is an active protein with various biological functions that participates in regulating glycolysis and plays a key role in regulating cell survival. The phosphorylation of PKM2 contributes to the formation of the PKM2 dimer and the translocation of PKM2 into the mitochondria after treatment with CP [55]. The regulation of PKM2 activity partially limits cell death. These natural antioxidant molecules provide the scientific basis to design clinical trials aimed at reducing the oxidative stress, cognitive alterations, and, probably, the CNS changes elicited by chemotherapy in cancer patients.

## 5. Conclusions

The results of this work show that 5-HIAA and ATPase increased during NAC and CP treatment. This event may have given rise to neuroprotection and augmented membrane fluidity, responsible for maintaining the electrochemical potential and sodium gradient across the plasma membrane. N-Acetylcysteine (NAC) is a drug with the ability to induce glutathionylation, and this portrays that it has the potential to mitigate neuronal and lung toxicity due to CP. Thus, the efficacy of a combined NAC and CP drug therapy appears to be a promising strategy for future chemotherapy in malnourished patients. We suggest that more research work should be carried out to thoroughly examine its neuroprotective mechanisms. Our study has some limitations that are important to mention. We did not try different concentrations of the drug combinations. However, the novelty of this study is that, differently from other studies performed with this cancer drug, it involves an important variable—protein deficiency—thus being the first study of its character in chemotherapy based on CP. In addition, this study measured the adverse effects of NAC on brain regions.

## Figures and Tables

**Table 1:** Glucose and triglyceride levels in the blood of animals with protein deficiency treated with NAC and NAC + CP.

	Glucose (g/dL)		Triglycerides (g/dL)	
Group	Mean	S.D	Mean	S.D
Control	177.67	20.73	112.17	23.16
NAC	169.17	19.53	131.33	25.87
Cisplatin	194.00	9.78	134.33	20.09
NAC + Cisplatin	152.00	12.46 ***	135.00	30.71

ANOVA: Glucose: *** *p* = 0.0009 vs. Cisplatin. Triglycerides: N.S.

**Table 2:** Lipid peroxidation (Tbars) levels in the brain regions of animals with protein deficiency treated with NAC and NAC + CP.

	Tbars µM Malondialdehyde/g Tissue					
	Cortex		Hemispheres		Cerebellum	
Group	Mean	S.D	Mean	S.D	Mean	S.D
Control	9.75	3.06	14.51	4.47	12.07	3.56
NAC	8.38	0.88 *	11.34	1.36	8.95	1.97
Cisplatin	6.36	0.45 *, **	8.50	1.26 ***	7.45	0.45 *
NAC + Cisplatin	6.57	1.18 **	7.70	0.75 *, ***	6.68	0.71 *

ANOVA: Cortex: ** *p* < 0.01 vs. Control; * *p* < 0.02 vs. NAC. Hemispheres: *** *p* = 0.0001 vs. Control; ** *p* < 0.01 vs. NAC. Cerebellum: * *p* = 0.04 vs. Control.

**Table 3:** Glutathione (GSH) levels in the brain regions of animals with protein deficiency treated with NAC and NAC + CP.

	GSH nM/g Tissue					
	Cortex		Hemispheres		Cerebellum	
Group	Mean	S.D	Mean	S.D	Mean	S.D
Control	439.08	152.11	374.10	137.17	468.29	147.44
NAC	481.76	111.31	331.50	74.05	273.93	91.97
Cisplatin	364.40	151.43	295.58	74.65	330.98	80.80
NAC + Cisplatin	235.74	104.92 **	389.84	99.50	322.70	68.09

ANOVA: Cortex: ** *p* = 0.01 vs. Control. Hemispheres: N.S. Cerebellum: N.S.

**Table 4:** H_2_O_2_ levels in the brain regions of animals with protein deficiency treated with NAC and NAC + CP.

	H_2_O_2_ µM/g Tissue					
	Cortex		Hemispheres		Cerebellum	
Group	Mean	S.D	Mean	S.D	Mean	S.D
Control	0.07	0.04	0.02	0.00	0.03	0.02
NAC	0.02	0.02 *	0.02	0.01	0.02	0.00
Cisplatin	0.03	0.02	0.03	0.01 *, ***	0.02	0.00
NAC + Cisplatin	0.03	0.02	0.01	0.00	0.02	0.01

ANOVA: Cortex: * *p* = 0.02 vs. control Hemispheres; * *p* = 0.02 vs. Control and *** *p* = 0.001 vs. NAC + Cisplatin. Cerebellum: N.S.

**Table 5:** Ca^+2^ and Mg^+2^ ATPase activity in the brain regions of animals with protein deficiency treated with NAC and NAC+ CP.

	ATPase µM Pi/g Tissue/min					
	Cortex		Hemispheres		Cerebellum	
Group	Mean	S.D	Mean	S.D	Mean	S.D
Control	465.38	187.74	894.59	206.13	518.11	115.99 *, ***
NAC	468.98	52.32	728.77	105.63	616.57	85.97
Cisplatin	439.19	62.71	749.82	41.32	608.58	83.09
NAC + Cisplatin	479.72	58.41	769.23	140.18	647.94	43.63

ANOVA: Cortex: N.S. Hemispheres: N.S. Cerebellum: * *p* = 0.03 vs. Cisplatin,* *p* = 0.02 vs. NAC, and *** *p* = 0.004 vs. NACC + Cisplatin.

**Table 6:** 5-HIAA levels in the brain regions of animals with protein deficiency treated with NAC and NAC+ CP.

	5-Hiaa mM/g Tissue					
	Cortex		Hemispheres		Cerebellum	
Group	Mean	S.D	Mean	S.D	Mean	S.D
Control	2.17	1.00	3.68	1.17	2.37	0.57
NAC	2.49	0.19 ***	3.34	0.32	2.41	0.32
Cisplatin	2.15	0.36	2.72	0.23 *	2.77	0.47
NAC + Cisplatin	2.24	0.30	2.87	0.36	2.11	0.58

ANOVA: Cortex: *** *p* = 0.007 vs. Control. Hemispheres: * *p* = 0.02 vs. Control. Cerebellum: N.S.

**Table 7:** Lung Ca^+2^ and Mg^+2^ ATPase activity of animals with protein deficiency treated with NAC and NAC + CP.

	ATPase µM Pi/g Tissue/min	
	Lung	
Group	Mean	S.D
Control	155.99	33.89
NAC	199.73	47.57
Cisplatin	164.89	22.36
NAC + Cisplatin	237.87	38.96 *, ***

ANOVA: *** *p* = 0.005 vs. control, * *p* = 0.01 vs. Cisplatin.

## Data Availability

All the data generated or analyzed during this study are included in the published article.
